# Unraveling a Molecular Adhesion Mechanism at Complex Polymer–Metal Interfaces

**DOI:** 10.1021/acsami.4c13314

**Published:** 2024-11-04

**Authors:** Lukas Kalchgruber, Michael Hahn, Kai A. Schwenzfeier, Martin Rester, Christian Weissensteiner, Laura L. E. Mears, Markus Valtiner

**Affiliations:** † Institute of Applied Physics, Vienna University of Technology, 1040 Vienna, Austria; ‡ CEST Centre for Electrochemical Surface Technology GmbH, 2700 Wiener Neustadt, Austria; § Institute of Applied Physics, Applied Interface Physics, Vienna University of Technology, 1040 Vienna, Austria; ∥ Institute of Chemical Technologies and Analytics, Vienna University of Technology, 1060 Vienna, Austria; ⊥ Berndorf Band GmbH, 2560 Berndorf, Austria; # Christian Doppler Laboratory for Surface and Interface Engineering, Institute of Applied Physics, Vienna University of Technology, 1040 Vienna, Austria

**Keywords:** adhesion, stainless steel, SFA, contact mechanics, hydrogen bonding, van der Waals forces, rough samples, surface treatment

## Abstract

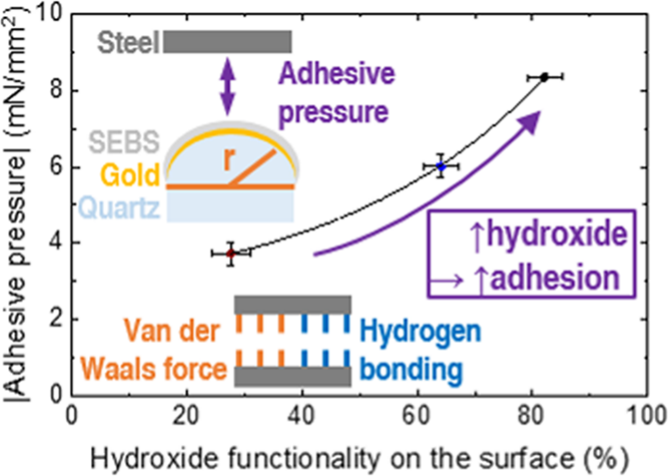

Controlling polymer–metal adhesion is critical in ensuring that materials can be cleanly separated during production processes without residue, which is crucial for various industrial applications. Accurately characterizing adhesion on industrial-grade surfaces is complex due to factors like surface roughness and actual contact area between surfaces and the polymer. In this study, we quantified the adhesive behavior of stainless-steel samples with varying surface treatments against a polymer using the surface forces apparatus (SFA) in reflection geometry, as well as X-ray photoelectron spectroscopy (XPS). We compared adhesive properties with the penetration depth of oxygen and the hydroxide-to-oxide ratio, which were modified by plasma and thermal treatments. Our results indicate that both treatment types enhance the deadhesive properties of the materials compared to native passive films, due to decreasing hydroxide functionality on the surface. Thermal treatment reduces adhesion further, due to an even lower hydroxide content, which reduces hydrogen bonding between the surface and polymer. Furthermore, we show that van der Waals forces, which depend on the density, have marginal to no influence on the adhesive behavior. This study not only advances our understanding of the factors influencing polymer–metal adhesion but also demonstrates the application of the SFA in reflection geometry for characterizing industrially relevant rough surfaces.

## Introduction

Effective polymer–metal deadhesion is essential for ensuring that materials can be separated cleanly and without residue in manufacturing processes. This is vital for numerous industrial applications, including roll-to-roll processes, or polymer casting, where deadhesion is essential. In these applications softer polymers adhering to rough metal surfaces are of interest. To quantify adhesion various methods can be used. These techniques often quantify how much force is needed to separate two surfaces of interest. A main challenge is that area normalization of adhesive forces is complex, especially for industrially relevant rough surfaces.^1^ The most commonly used semiquantitative method for the characterization of polymers in contact with various materials is the peel test.^2^ As an alternative, indentation tests can be used, their main field of use is the characterization of laminates.^3^ Furthermore, blister tests can be performed, where a liquid is injected beneath the coating to form a blister until it breaks.^4^ Finally, one method, which is not reliant on force measurements, is measuring contact angles. The adhesive behavior can be estimated by indirectly measuring the surface energy. Relating the surface energy to the adhesive behavior is however challenging since the measurements are performed with various liquids (e.g., water and diiodomethane), and not directly with the substances of interest.^5^

A system that offers the possibility to measure the adhesive force for industrially relevant rough surfaces directly, in an absolute way, would hence be very beneficial for tailored development of industrial tools. So far, it has been difficult to predict how different substrate surface treatments would change polymer release properties, and predictions relied on empirical knowledge. For a targeted tuning of deadhesion, two requirements have to be met. First, the adhesive behavior of industrially relevant materials, with varying roughness and surface composition, has to be quantified. Second, the surface of the material has to be characterized and related to adhesive characteristics. Relevant characterization parameters are the roughness, the penetration depth of oxygen and the type of species present at the surface. Altering the surface properties and subsequently characterizing their influence provides design options to optimize materials specifically for certain applications, i.e., for operating with required types of polymers.

The surface modifications can be achieved by numerous surface treatment methods. Two types of surface treatment are of particular interest to industry and in the context of this paper, namely, thermal and plasma treatment. Both treatments are intended to alter the surface and induce various changes. One of the common purposes is to increase the passive layer thickness and by that, increase the corrosion resistance.^6−9^ Compared to the commonly used thermal treatment, plasma treatment has many advantages including low operating costs, short treatment times and the technique is environmentally friendly. The disadvantages are, high equipment investment costs, determination of the effective dose and limitations to the number and the size of the samples.^10^

To quantify adhesive behaviors swiftly and directly in a modular approach, a direct and area normalized force measurement of a breaking contact is ideally suited. For this we adapted the surface forces apparatus (SFA) for operation with industrial-grade steel surfaces. The SFA is an optical technique based on multiple beam interferometry (MBI).^11−13^ Many adaptations have been made over the years to improve the problem-space accessible and to be able to operate the instrument in reflection geometry.^14−18^ The operation in reflection geometry was a key function for this study, since it allows the use of metals, as a nontransparent sample, directly in the SFA.^17−19^ Some recent examples for the applications of SFA are wettability characterization of asphaltenes,^20^ adhesion of Langmuir–Blodgett polymer layers,^21^ the behavior of ionic species in confinement,^22^ and concrete deterioration due to the alkali-silica reaction (ASR).^23^ The accessible parameters include oxide thicknesses, separation distances, contact radii and normal forces.^18^ Adhesion can be directly quantified as the normal force measured between the surfaces upon separation. Adhesive pressure can be calculated based on the direct visualization of the real contact area.

In detail, here stainless steel in direct contact with the polymer SEBS was probed. Different surface treatments (temperature and plasma) were applied to the stainless steel to change the surfaces and absolute adhesive pressures were measured. Thus, a reflection geometry version of SFA was required. To enable characterization of industrial-grade samples, a soft polymer was used as a fully compliant adhesion probe. The measured adhesive pressures were related to the interaction types present (van der Waals forces and hydrogen bonds). For this comparison, elemental and chemical analysis was performed by X-ray photoelectron spectroscopy (XPS),^24^ and sputter depth profiling was used to probe the penetration depth of oxygen.^25,26^ The analysis allows the determination of the hydroxide-to-oxide ratio on the surface, which alters the adhesion due to hydrogen bonding. The penetration depth of oxygen directly influences the van der Waals forces due to different densities of the bulk metal compared to the density of the surface region.^27^ The proposed complementary SFA and XPS measurements provide a way to predict the release properties for industrially relevant surface pretreatments.

## Methods and Materials

### Chemicals and Samples

Ethanol (≥99.9%), sulfuric acid (98%), hydrochloric acid (37%), and polystyrene-*block*-poly(ethylene-*ran*-butylene)-*block*-polystyrene (= SEBS, < 1% Antioxidant, av. *M*_w_ ≈ 89,000 g mol^−1^ by GPC) were ordered from Sigma-Aldrich. Cyclohexane (>99.9%) and hydrogen peroxide (50%) were purchased from Carl Roth GmbH. For the preparation of all solutions Milli-Q water (Merck Millipore purification system, resistivity of 18.2 MΩ·cm, TOC ≤ 2 ppb) was used.

The analyzed samples were prepared from stainless steel from Berndorf Band GmbH. The stainless steel is composed of 75.2 wt % iron, 15.2 wt % chromium, 5.0 wt % nickel, 0.7 wt % manganese, 3.2 wt % copper, and some low concentration elements. This steel was used as the reference sample (*R*). Furthermore, the samples were treated with either temperature (*T* samples) or with a plasma (*P* samples). The following conditions are a representative selection of parameters used in industry which provide a variety of surface changes. The specific parameters for the five *T* samples were: *T*1 (505 °C, 90 min), *T*2 (300 °C, 10 min), *T*3 (300 °C, 30 min), *T*4 (300 °C, 120 min), and *T*5 (400 °C, 30 min). For the *P* samples the energy input was varied by changing the distance and the feed of the plasma nozzle, leading to different energy inputs per area: *P*1 (low), *P*2 (high), *P*3 (medium) and *P*4 (medium). Each *T* and *P* sample was measured once, to obtain statistical information the sample sets were averaged (*T*_av._ and *P*_av._). The reference sample was measured seven times and the values were averaged (*R*_av._).

### Preparation

For the measurements, squares (1 cm × 1 cm) of the various stainless-steel samples were cut out. Each sample piece was cleaned in absolute ethanol for 10 min in an ultrasonic bath before each measurement. Afterward the samples were dried with a nitrogen gun. For the preparation of the SFA disks, they were cleaned with aqua regia and piranha solution to remove the metal layers and clean the disks before metal deposition (as described below in the SFA section) and the spin-coating process.

### Confocal Microscopy

A confocal microscope, a μsurf explorer from NanoFocus, with the software μsoft control, was utilized. For all pictures a magnification of 20x was used. The confocal microscope was used on the one hand to check the quality of the coating and on the other hand to evaluate the roughness of the stainless-steel specimen as described in the next section. Furthermore, confocal microscopy was utilized to evaluate the sputter rate of the XPS sputter analysis.

### Surface Forces Apparatus

To quantify the adhesion of a polymer with a stainless-steel surface, a home-built surface forces apparatus (SFA) was utilized. The setup has an depth resolution limit of 0.02–0.04 nm, a lateral resolution of 0.5–2 μm and consists of several optical parts as published by Schwenzfeier et al.^18^ To briefly summarize, the SFA was used in reflection geometry, which is where light is reflected between an opaque mirror-like sample, steel, and a semitransparent mirror on the apposing curved surface with an optical spacer in between, forming a cavity. The interference pattern can be captured either spectroscopically or in the form of Newton’s rings, which was primarily used here, using a single wavelength filter (580 nm, Thorlabs Inc.). The optical path^18^ and the specific strain gauge information have been published previously.^28^

**Figure 1:**
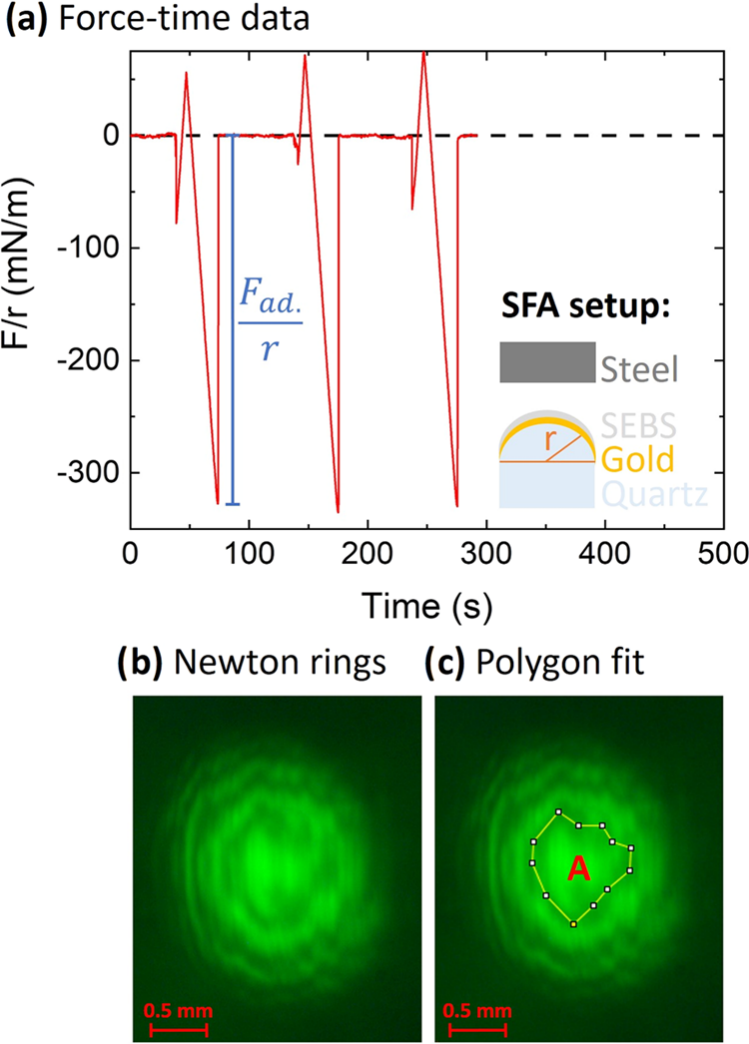
(a) Progression of force over time data from the strain gauge within the SFA setup. For the *y*-axis the measured force, referenced to the disk’s radius of curvature (*r* = 2 cm), was plotted. Furthermore, the experimental setup is depicted as scheme. Additionally, the relevant variables are defined. (b) Newton rings for contact area (*A*) evaluation. (c) Polygon fit highlighted. Each picture has a size of 0.25 × 0.30 cm.

In the experiment (schematic depicted in Figure 1), the differently treated steel samples are brought into contact with a polymer. For that, an SFA disk, which is a fused silica optical disk in the shape of a cylinder capped with a spherical dome with a curvature radius (*r*) of 2 cm, is needed. First, the optical disk is coated with a semitransparent reflective layer. This is deposited on the curved surface by physical vapor deposition (PVD). Specifically, ≈5 nm titanium is deposited at <9.8 × 10^–3^ mbar, followed by ≈40 nm of gold at ≈5 × 10^–6^ mbar. Second, the polymer of interest was spin-coated onto the gold surface using a self-built spin-coater. The requirement for this coating was to form a several μm thin, homogeneous and smooth layer to be usable as both the compliant, adhesive surface and the optical spacer for the SFA experiment.

Afterward, the steel sample and the polymer are brought together in a particle free contact in the SFA. The following two processes were used alternately. First, a triangular signal with an amplitude of 400 mV and a frequency of 10 mHz was applied to the piezo to produce a speed of 40 nm/s when moving freely. This leads to a constant approach velocity for all runs. In the next step the two surfaces were moved away from each other with constant voltage (≈400 mV) to a large distance (approximately 4 μm) which is well beyond the point where a force can no longer be detected between the surfaces. These steps were repeated, leading to periodically contacting the sample with subsequent separation of the surface. The resulting forces were measured via a strain gauge and are depicted in Figure 1a. This process was repeated three times and the measured adhesive forces (*F*_ad._) were averaged. In Figure 1a, the data were referenced to the radius of curvature of the SFA disk, *r*. Data from the strain gauges was converted to force via a calibration with known masses and using a script written by the authors in Python.

To calculate the effective absolute adhesive pressure (*p*), the data were referenced to the contact area (*A*). For that the Newton’s rings (Figure 1b) were used. The contact area was estimated, using the ImageJ analysis software package, by fitting polygons (Figure 1b) three to five times and then averaged to minimize operator effects.^29^ Furthermore, the roughness of the steel specimen was included. For this, the samples were analyzed by confocal microscopy before the SFA measurement. To obtain the average roughness factors (*f*), lines at 20, 40, 60, and 80% of the picture height were taken and averaged. These roughness factors are defined as the ratio between the length of the extracted profiles and the width of the image. The average roughness factors of the respective sample sets were *f*_R_ = 17 ± 3, *f*_P_ = 16 ± 3 and *f*_T_ = 14 ± 3. No major changes in the surface roughness due to the surface treatment are visible. The area from the Newton’s rings was multiplied with the calculated roughness factors. This leads to roughness corrected areas (α): α_R_av.__ = (11 ± 2) × 10^–3^ cm^2^, α_P_av.__ = (12 ± 2) × 10^–3^ cm^2^ and α_T_av.__ = (18 ± 4) × 10^–3^ cm^2^. The adhesion did not vary significantly with small changes in the compression force which was kept approximately constant across all measurements (see Figure 1a) for a typical plot. The ratio of the maximum compression force to the *F*_ad._ was kept low at approximately 0.16.

### Polymer

As counterpart to the rough steel surface a compliant polymer, SEBS, was used. The only limitation is that the coating needs to be well attached to the gold film and its thickness has to be controlled in order to act as the optical spacer in the SFA. For SEBS this attachment was achieved by spin-coating with a self-built setup. The quality of the coating was validated with confocal microscopy to guarantee a smooth and intact coating. In this work we chose the polymer SEBS due to its advantageous properties, including transparency, softness, elasticity, stickiness and the solvent solubility for spin-coating. These properties enable SEBS to conform to surface roughness and establish a close contact to the stainless-steel samples.^30^ Furthermore, SEBS has a pi-system that is known to form hydrogen bonds when the counterpart has hydroxide groups present.^31,32^ For studying hydrogen bonding, a pi-system has the advantage that the hydrophilicity of the polymer is not increased and, therefore, water from the atmosphere does not alter the adhesive process significantly or induce capillary formation and forces. A suitable solution concentration for the spin-coating of SEBS is 9 wt % in cyclohexane.^33^ Due to the excellent solubility of SEBS in cyclohexane, the coating made in this step can easily be removed and reapplied.

### Spin Coating

For the spin-coating a self-built spin-coater was utilized. For this a 2600 kV brushless DC drone motor was used. For rotation speed control an Arduino circuit board was coded. To measure the rotation speed, and have the option to keep it constant, a Tachometer PCE-DT 50 from PCE instruments was bought. The procedure of spin-coating starts with applying 4–6 drops of SEBS in 9 wt % cyclohexane solution to the disk. During which the disk rotated with constant ≈3000 rpm. To reduce the number of particles on the coating, the cyclohexane solution was filtered with a 0.2 μm polypropylene membrane syringe filter (VWR International) prior to the process.

### X-ray Photoelectron Spectroscopy

The X-ray photoelectron spectroscopy (XPS) measurements were carried out with an Axis Supra spectrometer from Kratos Analytical at the CEITEC Nano Research Infrastructure. For those measurements Al Kα X-rays were used from an aluminum anode. The spot size of the beam was 700 × 300 μm^2^. XPS was used to characterize the steel samples, namely, *R*, *P*, and *T* samples, without any contact to SEBS. The dwell time was 260 ms. Since the samples were conductive no charge neutralization was necessary. The samples were sputtered with an ion gun with 20 keV Ar500+ ion clusters. Argon 5.0 purity was used for this. For the transition of the sputter steps to a sputter depth, one *T* sample (*T*1) was sputtered until a crater, which is deep enough to successfully be analyzed with confocal microscopy, was produced (depth ≈ 1.2 μm). Based on that evaluation, a sputter rate of 1.96 nm/min was determined and used to estimate the sputter depths for all samples.

To evaluate the data CasaXPS was used.^34^ The steps taken for data evaluation of the oxygen spectra are as follows. First, the binding energy scale was calibrated, with the C 1s peak shifted to 284.8 eV.^35^ Second, the photoemission peaks were fitted and quantified after subtracting a Shirley-type background.^36^ For the O 1s spectra, three peaks (M=O, M–OH and adsorbed water) were used to fit the experimental data as suggested by Grosvenor et al.^37^ For the information about the penetration depth of oxygen, the visibility of the metallic iron compound was tracked. The metallic compound is present at lower binding energies compared to the various oxide species.

## Results and Discussion

For industrial applications one key question is how the adhesive interaction between a metal and a polymer can be quantified, since it is often of interest to separate these two materials from each other without leaving any residue behind, that is the release of the polymer from the metal. For the metallic part stainless-steel samples with differently treated surfaces were used. They were divided into different sets, namely the thermally treated samples (*T* samples), samples treated with plasma (*P* samples) and the reference sample (*R*), which had only the native passive layer on-top. For the polymer, which was spin-coated onto an SFA disk, SEBS was used. The contact within our system was adhesive as the visual inspection after the SFA measurement did not show SEBS residue on the stainless-steel sample.

### Adhesive Pressure

**Figure 2:**
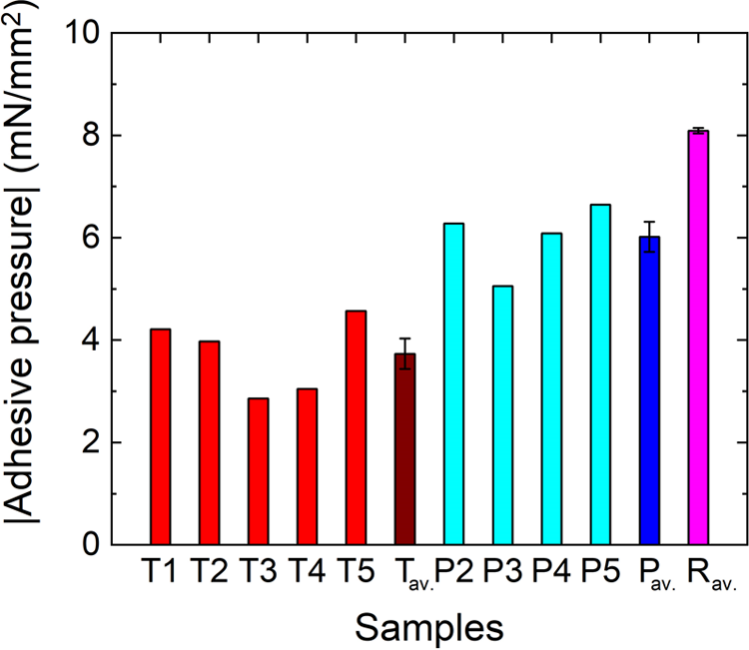
Bar chart of the absolute adhesive pressure. The measured samples are shown on the *x*-axis. Additionally, the average values of the sample sets *T*_av._, *P*_av._ and *R*_av._ are depicted, including their error bars. A clear trend is visible (*p*_R_av.__ > *p*_P_av.__ > *p*_T_av.__) in the measured data.

To quantify the adhesive pressure reflection geometry SFA measurements were performed. The differently treated steel samples were brought in direct contact with SEBS, and moved apart, cyclically, within the SFA setup. The resulting absolute adhesive pressure (*p*), is depicted in Figure 2.

#### Difference between the Sample Sets

To validate the statistical differences between the sample sets (*R*_av._, *P*_av._ and *T*_av._), two sample *t* tests, assuming equal variances based on Levene’s test, were performed.^38,39^ The Levene’s test was used to compare the variances of the *T* and *P* sample sets (critical value = 9.1, test statistic = 1.3) and to compare *P* and *R* (critical value = 8.9, test statistic = 0.35), and led to the result that the variances are the same. The statistical evaluation leads to the result that plasma treated samples on average show significantly more adhesion to the polymer than the thermally oxidized ones (critical value = 2.4, test statistic = 4.7). Furthermore, *R* shows significantly more adhesion compared to the *P* samples (critical value = 2.3, test statistic = 5.5). Leading to the trend of *p*_R_av.__ > *p*_P_av.__ > *p*_T_av.__. The confidence level of this statistical evaluation is over 99%. This shows that SFA in reflection geometry can be utilized to analyze and differentiate between differently treated rough stainless-steel samples by their adhesive behavior with the polymer SEBS.

The differences in the adhesive behavior of the differently treated stainless-steel samples, have their origin in the intermolecular interactions. The adhesive behavior can be altered when these intermolecular forces are changed. This can be achieved, for example, by adapting the penetration depth of oxygen which in turn would influence the van der Waals forces. Another option would be altering the hydroxide-to-oxide ratio on the surface, leading to a different strength and number of hydrogen bonds.

### Intermolecular Interactions

For our system, stainless steel in contact with the polymer SEBS, two different interactions are possible. First, van der Waals forces can be present. Second, hydrogen bonds can affect the adhesion. Other interactions are not possible as permanent charges are neither present in the polymer nor in the passive layer of the steel, no medium other than air is present and covalent bonding will not occur. The interaction energies both from the measurements and the theoretical contributions require a number of assumptions, and for such a complex contact geometry, they can only be considered as estimates. However, as each data set is treated the same way, the trends and comparisons between the samples are consistent.

#### Van der Waals Forces

Assuming different penetration depths of oxygen, due to different surface treatments, would lead to different van der Waals forces due to their dependency on the density as stated by Israelachvili among others (eqs 1a and 1b).^27^
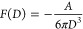
1a
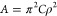
1b

**Figure 3:**
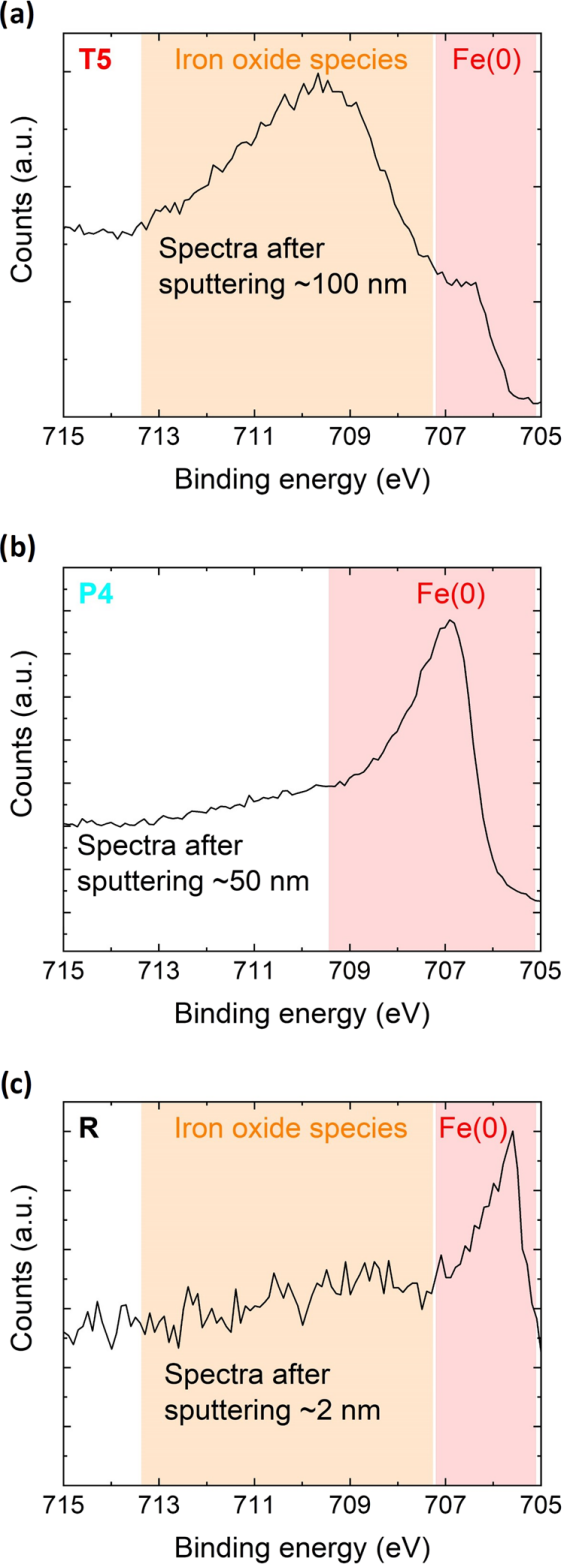
Fe 2p spectra of the three different sample types, one representative example of each data set given. (a) Thermally treated sample (*T*5), (b) plasma treated sample (*P*4) and (c) reference sample (*R*). The spectra are given at different sputter depths. The reference sample data has a dominant metallic peak after sputtering ≈2 nm and is completely metallic after ≈5 nm. For the *P* samples, at the beginning mainly oxide species are present, while the spectra become purely metallic after sputtering approximately 50 nm. The *T* samples showed purely oxide species until 100 nm was sputtered away, where the process was stopped when metallic species started to become visible.

Equations 1a and 1b show that the van der Waals forces depend on the distance between two flat surfaces (*D*) and the Hamaker constant (*A*), which in turn depends on the density of the material (ρ) and the particle–particle interaction (*C*). With the metallic iron having a higher density (ρ_Fe_ = 7.87 g/cm^3^) compared to the iron oxide species (ρ_Fe_2_O_3__ = 5.24 g/cm^3^) it can be anticipated that a higher penetration depth of oxygen leads to lower van der Waals forces. To obtain the penetration depth of oxygen (*d*), sputter XPS analysis was performed. Exemplary spectra of the three different sample types are depicted in Figure 3.

The XPS measurements showed that the penetration depth of oxygen (*d*) of the different samples varies (*d*_R_av.__ ≈ 5 nm, *d*_P_av.__ ≈ 50 nm, *d*_T_av.__ > 100 nm). The reference sample is already fully metallic after removing 5 nm of the surface. As illustrated in Figure 3, these depths were determined by the appearance of the metallic Fe(0) peak in the XPS sputtering measurements.

To quantify the influence of the van der Waals forces and how they contribute to the adhesive pressures shown in Figure 2, intermolecular force estimations were made. The estimations of the van der Waals forces are based on the Hamaker constant and the flat on flat geometry suggested by Israelachvili (eqs 1a and 1b).^27^ Our setup, a planar steel surface in contact with a spherical SFA disk would suggest to use the JKR approach for a flat on sphere geometry. The JKR approximation uses the Derjaguin approximation applied to the flat on flat geometry, to account for the contact area of a smooth round contact, rather than determining the energy of adhesion as a surface density. Since our contact area is both rough and not a uniform circular shape (Figure 1), we initially compare our energy densities based on the assumption of a flat on flat geometry. The alternative JKR approach and other potential corrections to the estimation of energy from the contact are discussed further below.

For the Hamaker constant, literature values were chosen (*A*_Fe_ = 562 × 10^–21^ J and *A*_Fe_2_O_3__ = 250 × 10^–21^ J).^40−42^ To calculate the energy of the van der Waals forces, the force was integrated over different depth intervals. As a starting point of the integration 0.33 nm was chosen, which is the approximate length of a hydrogen bond, since the used formula does not converge to zero due to the Pauli limitation. The integral was split into two integrals, with the first one using the Hamaker constant of the oxide and the second integral using that of metallic iron. The boundary between the oxide and metallic integrals used was the oxygen penetration depth (*d*). Contributions from beneath 100 nm were neglected. This calculation is shown in eq 2.
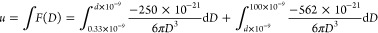
2

This evaluation leads to the energy densities (*u*). The estimations showed that only a marginal difference between the *P* and *T* samples is present (*u*_P_av.__ = (−6.0867 ± 0.0001) × 10^–2^ J m^–2^ and *u*_T_av.__ = (−6.08943 ± 0.00003) × 10^–2^ J m^–2^). The difference in the absolute adhesive pressure (Figure 2) between the *P* and *T* samples is therefore not due to van der Waals forces. This is due to their low range of effect. However, the reference sample (*u*_R_av.__ = (−6.12 ± 0.01) × 10^–2^ J m^–2^) showed a slightly higher absolute value than the other sample sets, which might lead to a slightly higher adhesive pressure for the native sample.

#### Hydrogen Bonding

**Figure 4:**
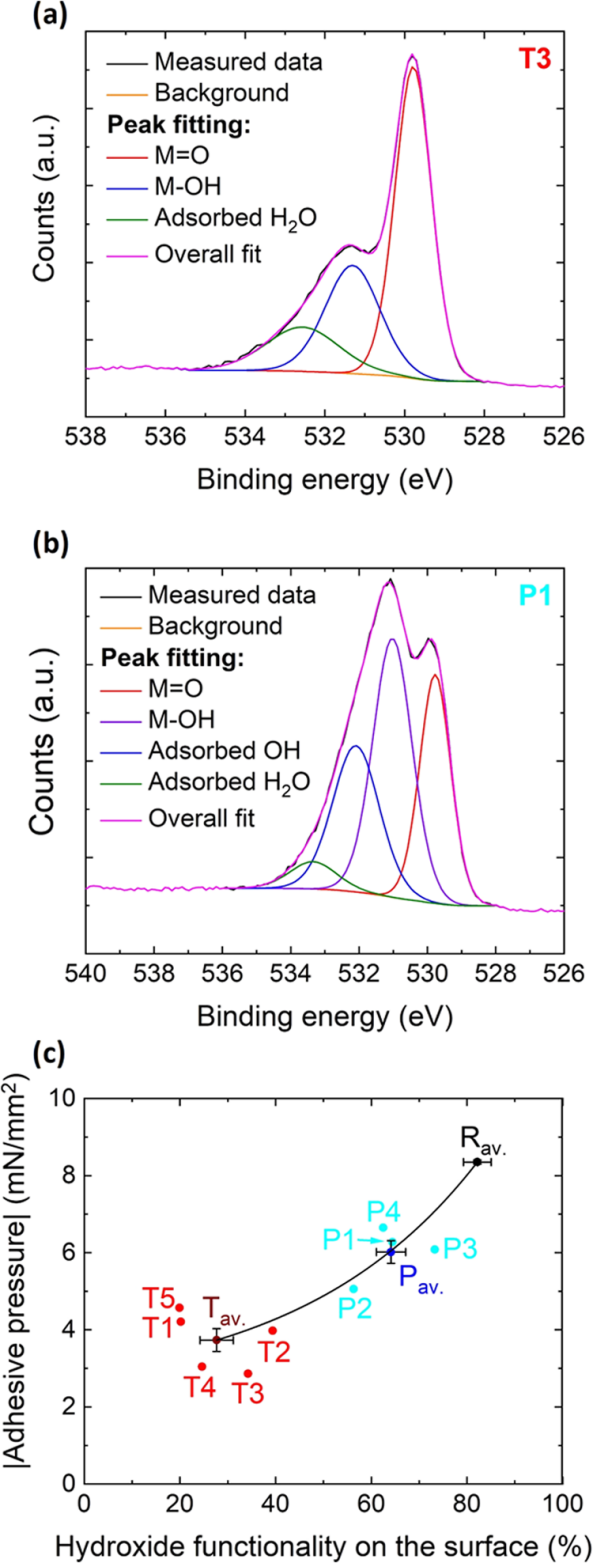
O 1s spectra of the surface of (a) thermally treated sample (T3) and (b) plasma treated sample (P1). Peak fitting indicated in different colors. (c) Percent of hydroxide functionality vs the measured absolute adhesive pressure from the SFA experiment. The sample sets can be differentiated and a trend is visible. The solid line is a guide to the eye to highlight the trend.

The second form of interaction possible in our system, beside van der Waals forces, are hydrogen bonds. Two different sources of hydrogen bonds are present. First, the hydrogen from the hydroxide group of the steel sample can be the proton donor with the proton acceptor being the π-system of the polymer. Second, the C–H of the polymer can act as proton donor with the oxygen of the oxide group of the steel sample as proton acceptor. Since the samples had different surface treatments, it is expected that their surface hydroxide-to-oxide ratio is different. To quantify this additional XPS measurements were performed. The resulting data was plotted versus the absolute adhesive pressure in Figure 4. It is evident that the sample sets can be differentiated and that significant variation of OH/O ratio is present. Lower hydroxide functionality on the surface leads to lower absolute adhesive pressures and vice versa.

The trend shows that the temperature treatment led to less hydroxide formation on the surface compared to the plasma treatment. Both treatments reduced the amount of hydroxide on the surface, however, by differing amounts owing to differences in the treatment atmosphere. Thermal treatment is performed in a very dry atmosphere, less water in the atmosphere means that less hydroxide functionality is formed on the surface. On the contrary, the plasma treatment is performed in an atmosphere with comparatively higher humidity, therefore, more water is present and because of that comparatively more hydroxide can form on the surface.

As previously for the van der Waals forces, estimations of the strength and contribution of the hydrogen bonding to the adhesive pressures, shown in Figure 2, were performed. For this calculated H-bond energies of gas-phase dimers were used.^43^ For the first type, the interaction of MeOH with a benzyl group was chosen as it is similar to the interaction in our system (≈2.8 kcal mol^–1^). The approach from Malone et al. used searches through the Cambridge structural database, ab initio molecular orbital and semiempirical calculations.^44^ For the second variant of the hydrogen bond, CH_4_ with H_2_O was used (≈0.3 kcal mol^–1^). The approach of Gu et al. was also based on ab initio calculations.^45^ The latter one (CH_4_ - H_2_O) has a lower value, therefore it was neglected in further estimations.

Based on the surface density of metallic iron atoms (*N*_Fe surface_ = 1.9 × 10^15^ atoms cm^–2^), the number of surface atoms (*N*) was calculated, assuming a fully metallic iron surface. The number of hydroxides on the surface (*N*_OH surface_ = 1.1 × 10^15^ atoms cm^–2^) was converted from *N*_Fe surface_. The transition factor depends on the crystal structure of Fe(OH)_2_, which crystallizes in the cadmium hydroxide structure, where 16 metal atoms form 9 hydroxide groups. Further, the hydroxide ratio from the XPS measurements (Figure 4) and the Avogadro constant (*N*_A_) were used to estimate the moles of hydroxide per area (*n*) on the surface for each sample set (*n*_R_av.__ = (148 ± 5) × 10^–11^ mol cm^–2^, *n*_P_av.__ = (116 ± 5) × 10^–11^ mol cm^–2^ and *n*_T_av.__ = (50 ± 6) × 10^–11^ mol cm^–2^). Finally, to obtain the energy densities of the hydrogen bonds (*u*), *n* was multiplied by the literature value of the MeOH to benzyl interaction.^44^ It is evident that the energy density is the highest for the reference sample (*u*_R_av.__ = (174 ± 6) × 10^–3^ J/m^2^), while the *P* and *T* samples showed lower values (*u*_P_av.__ = (136 ± 6) × 10^–3^ J/m^2^ and *u*_T_av.__ = (58 ± 7) × 10^–3^ J/m^2^). The hydrogen bond strength estimations have the following trend *T*_av._ < *P*_av._ < *R*_av._ (Figure 4). The trend shown in Figure 2 is in accordance with the hydrogen bond strength estimation.

#### Comparison and Geometric Considerations

An initial comparison of the energy densities indicates that the van der Waals contribution, *u*_VdW_ = 10^–2^ J m^–2^ is weaker than the hydrogen bonding energy density, *u*_H-bond_ = 10^–1^ J m^–2^. When the energy density of the measured data is derived, once the true area with the roughness factor is included it changes the energy density slightly from the value without roughness *u*_meas., no rough._ = 10^–5^ J m^–2^ without roughness to *u*_meas., rough_ = 10^–6^ J m^–2^ with the roughness corrected area. Both, however, are smaller than the energy densities calculated for the van der Waals and hydrogen bonding. The most likely reason for this difference is due to estimating maximum values. For example, it was assumed in the estimation that 100% of the hydroxide functionalities form hydrogen bonds. Furthermore, deviations could be due to measurement inaccuracies, discrepancies in the literature values or an overestimation of the contact area due to the roughness factors. Comparing the magnitude of the hydrogen bonding and the van der Waals forces leads to the conclusion that the hydrogen bonds dominate the adhesive behavior. This result supports the conclusions derived above.

If instead of a flat on flat geometry, a sphere on flat, JKR approximation, is assumed there are formulas to predict the energy based directly on the radius of the contact at zero force.^27^ Since our contact is not perfectly circular (Figure 1), it would be necessary to assume a perfect circle to convert our contact area into a radius. Using this conversion, without the roughness factor, leads to an energy in the region of ×10^–10^ J and therefore a density of ×10^–3^ J m^–2^.

When the JKR approach is used, an elastic half-space is also assumed. A thin polymer film on a rigid surface, as used in our experiments, requires a correction to the elastic modulus.^46^ First, the formula can be simplified when the Poisson ratio is roughly 0.5, which is true for SEBS.^47,48^ Afterward, to perform this correction we need the confinement ratio (contact radius divided by film thickness). Since the procedure of applying the SEBS to the disk was always performed the same way, the same layer thicknesses can be expected, leading to the conclusion that the trend, in the magnitude of the energies between the different surface treatments, would not be changed since the influence would be a constant factor.

A further modification factor, that could be included, in the theoretical calculations is dissipation.^49^ As stated by Shull et al., the contact perimeter can be viewed as a crack and because of that the tendency of a material toward crack formation relates to the likelihood for dissipation processes.^46^ SEBS shows no tendency for crack formation as it is frequently used as an additive to reduce crack formation.^50,51^ Further dissipation would only influence the absolute value and not the presented trend as the polymer and the film thickness were not varied within our experiments.

The most nontrivial correction that we will discuss, that could be applied, is for the roughness of the interface. As stated in the Methods section, SEBS was chosen due to its ability to adapt to the roughness of the surface. Owing to that, the contact area was generally defined as the geometrical area multiplied with roughness factors from the confocal microscope measurements. There is a risk this might overestimate the real contact area. Furthermore, roughness could influence the adhesive behavior in a more complex way than would be included using an estimate based on direct scaling of the area. However, since the surface roughnesses throughout our experiments were similar, within the error of the calculation they were the same, roughness should not change the trend in surface energy presented. Alternative models, such as the Persson-Tosatti model, to predict more complex roughness changes could be used.^52,53^ These again bring further assumptions into what would already be an estimate (owing to the nonuniform contact circle) if the JKR approach is applied.

For completeness all values were recalculated into the energy (*W*). To do that, the energy densities (*u*) of the van der Waals and hydrogen bond estimations were multiplied with the roughness corrected areas (α) of the Newton ring evaluation (see section: Methods and Materials). Furthermore, another estimation was performed for the recalculation of the measured adhesive pressure (Figure 2). For that, the distance over which the polymer is estimated to detach from the steel surface (1 nm), as suggested as a sensible cutoff distance for soft matter interactions by Israelachvili.^27^ While the magnitude of the measured data was 10^–11^ J, the magnitude of the van der Waals forces (10^–8^ J) and of the hydrogen bonding (10^–7^ J) are higher. Thus, there is no change to the assertion made above, from the energy densities, that the hydrogen bonds dominate the adhesive interactions and that the assumptions used to estimate the values will not change the trend in the data: that the greater the hydroxide functionality on the surface the greater the adhesion energy because it derives from the hydrogen bonding.

## Conclusions

In this work we employed SFA and XPS testing on differently treated stainless-steel samples to quantify adhesion properties and evaluate the origin of intermolecular forces contributing to adhesion.

We successfully quantified the adhesive behavior of a rough steel sample with SFA measurements in reflection geometry. To enable this new analysis approach the limitations of the technique were circumvented by adapting the methodology. The key adaptations were Newton ring polygon fitting to quantify the contact area, roughness corrections, optimizing the use of a deformable polymer film as the optical spacer layer and additional XPS testing for further information.

To further prove the effectiveness of our new analysis approach, different surface treatments were used to alter the steel samples and their effects on the adhesion were determined. The sample sets could be differentiated from each other, based on the underlying molecular adhesion mechanism.

Specifically, absolute adhesive pressures for differently treated steel samples were determined by SFA in reflection geometry. The sample, which had no surface treatment and therefore only the native passive layer present, showed the highest absolute adhesive pressure. By utilizing SFA in reflection geometry we showed that the release properties are improved when the sample is treated with plasma and are improved even more when thermally treated, compared to the native, reference sample.

It was shown. by complementary XPS measurements, that the surface treatments alter the penetration depth of oxygen. The temperature (*T*) treatment increased the penetration depth of oxygen more than the plasma (*P*) treatment. The increase of the penetration depth of oxygen showed only a little effect on the release properties compared to the reference sample. A further increase, which was present from *P* to *T* samples, had no further effect. Additionally, the differences in the hydroxide-to-oxide ratio on the surface were demonstrated. While the reference sample had a high hydroxide ratio on the surface, treating it with plasma or temperature decreased the hydroxide ratio significantly. The temperature treatment decreased the hydroxide ratio more than the plasma treatment. In our system, steel with SEBS, the release properties were improved when the hydroxide ratio on the surface was reduced. Additionally, estimation of the order of magnitude of the two present interactions showed that the visible trend of the absolute adhesive pressure, for the differently treated samples, is mainly due to hydrogen bonding. The van der Waals force’s magnitude is roughly 1 order of magnitude lower and therefore, has less impact on the release properties. To conclude, SFA in reflection geometry can be used successfully for a “real life” sample system to directly quantify the adhesion between a wider range of materials than previously explored. This new analysis approach leads to new opportunities to measure and characterize nonideal samples.

## Data Availability

The raw and processed data required to reproduce these findings are available from the corresponding author via www.repositum.tuwien.ac.at upon reasonable request.
